# An Estimation Method of Continuous Non-Invasive Arterial Blood Pressure Waveform Using Photoplethysmography: A U-Net Architecture-Based Approach

**DOI:** 10.3390/s21051867

**Published:** 2021-03-07

**Authors:** Tasbiraha Athaya, Sunwoong Choi

**Affiliations:** School of Electrical Engineering, Kookimin University, Seoul 02707, Korea; athayatasbiraha@kookmin.ac.kr

**Keywords:** arterial blood pressure (ABP), photoplethysmogram (PPG), deep learning, U-net, continuous, non-invasive

## Abstract

Blood pressure (BP) monitoring has significant importance in the treatment of hypertension and different cardiovascular health diseases. As photoplethysmogram (PPG) signals can be recorded non-invasively, research has been highly conducted to measure BP using PPG recently. In this paper, we propose a U-net deep learning architecture that uses fingertip PPG signal as input to estimate arterial BP (ABP) waveform non-invasively. From this waveform, we have also measured systolic BP (SBP), diastolic BP (DBP), and mean arterial pressure (MAP). The proposed method was evaluated on a subset of 100 subjects from two publicly available databases: MIMIC and MIMIC-III. The predicted ABP waveforms correlated highly with the reference waveforms and we have obtained an average Pearson’s correlation coefficient of 0.993. The mean absolute error is 3.68 ± 4.42 mmHg for SBP, 1.97 ± 2.92 mmHg for DBP, and 2.17 ± 3.06 mmHg for MAP which satisfy the requirements of the Association for the Advancement of Medical Instrumentation (AAMI) standard and obtain grade A according to the British Hypertension Society (BHS) standard. The results show that the proposed method is an efficient process to estimate ABP waveform directly using fingertip PPG.

## 1. Introduction

Blood pressure (BP) monitoring and management in the normal range is vital to a healthy life. Fluctuation in BP has a strong correlation with several organ injuries in the case of hypertension [1]. Hypertension is identified as one of the major risk factors for ischemic heart disease. According to the World Heart Federation, about 50 percent of ischemic strokes are caused by hypertension [2]. Furthermore, it also increases the risk of hemorrhagic stroke, heart failure, heart attack, and chronic kidney disease [2,3]. In the last 15 years, these diseases have remained the leading causes of death globally [4]. So, appropriate control of BP is the basis of both primary and secondary ischemic heart disease prevention [5].

Although regular BP monitoring is important, there is an obvious antipathy towards BP measurement. The strong reason behind it can be the measurement process. Traditionally and most commonly, a mercury-tube sphygmomanometer with a cuff is used to measure BP. The measurement result is expressed in terms of systolic BP (SBP) and diastolic BP (DBP). But this measurement technique is discontinuous. So, many hypotensive cycles may be missed, that need more accurate and frequent detection. Moreover, it takes a long time to take readings using these devices, which is cumbersome. On the other hand, there is an invasive continuous BP measurement method, which is also known as direct BP measurement or invasive ABP. A catheter is inserted into an artery to conduct real-time BP monitoring. It can complete BP estimation in every cardiac cycle and monitor BP changes more precisely. Therefore, it is recognized internationally as the gold standard of BP monitoring methods [6]. But this approach is used only for critical patients and it has the risk of a range of complications, including infection [7].

Due to the importance of regular checking of BP and advancement in wearable sensor technology, many BP measurement methods have been proposed including the vascular unloading technique, the tonometry method, and pulse transit time (PTT)-based method. In the vascular unloading technique, which is also called the volume clamped method, a cuff is placed over a finger to measure blood volume change using a light source and detector by increasing and decreasing cuff pressure in an interlocking control loop [8]. But using continuous pressure for a long time is harmful and uncomfortable. The tonometry method [9] uses a sensor to measure palpation on the radial artery and measure continuous, non-invasive ABP. But tonometry method needs precise positioning and it is extremely sensitive to movement relative to the accessed artery [10]. It is not a popular approach at present due to its artifacts and users’ discomfort [11]. PTT is the time taken by a blood pulse to propagate from the heart to a peripheral site and it is inversely related to BP. Most of the PTT-based methods focus on the combination of two signals, photoplethysmogram (PPG) and electrocardiogram (ECG). In [12], an ECG and PPG signal feature-based BP estimation process was discussed. The feature set used in the study also included the PTT, that is the time difference between the R wave of ECG and systolic point of PPG. However, much research has stated that the R wave of ECG is not reliable to measure the beginning of a pulse, and the measuring equipment for ECG data acquisition is very complex and expensive, the sensors are also hard to find. In [13], a combination of PPG and seismocardiogram was applied in a wristwatch for SBP and DBP calculation. The device needed a high-resolution three-axis accelerometer sensor pressed to the sternum to measure heart vibrations. It is really difficult to develop and implement a very accurate accelerometer sensor and to take the measurement in a wired wearable-device setup. The authors used the Moens–Kortweg equation to calculate PTT from PPG and SCG with two subject-specific parameters. However, both of the parameters would differ for each subject which makes the overall BP estimation process troublesome. Besides, two different sensors were used to capture PPG and SCG. So, calibration between the two signals for individual sensors was needed to match the time difference.

Reviewing the difficulties of using a combination of signals, and as PPG is the most convenient signal to be used in wearable devices, researchers are trying to measure BP using only the PPG signal [14,15]. In [14], a total of 22 spectral and morphological features were extracted from PPG signals to characterize the signal to determine SBP and DBP output. The study [15] also used 8 features of pre-processed PPG signals as the input for different machine learning methods and the output was SBP and DBP. But different feature selection problem also persists in these studies. The work in [16] used preprocessed raw PPG signal windows with the first and second derivatives as the input of their modified ResNet-GRU-based network to predict SBP and DBP. However, this ResNet-GRU-based model is computationally expensive as the learning efficiency of gated recurrent unit (GRU) is low and converges slowly [17]. Similarly, another research work [18], used a convolutional neural network (CNN) model to get SBP and DBP output giving a similar preprocessed raw PPG signal window with the first and second derivatives as the input to the network. But their method does not satisfy the Association for the Advancement of Medical Instrumentation (AAMI) standard error range.

In this paper, we propose a method of estimating continuous and non-invasive ABP waveform using modified U-net deep learning architecture. Our method only needs the raw PPG signal to estimate the ABP waveform. And from the estimated ABP waveform, SBP and DBP can be estimated using a standard peak detection algorithm [19]. To the best of our knowledge, this is the first ABP waveform estimation method using PPG only. ABP waveform can provide additional information to the doctors more than just SBP and DBP values [20]. The ABP waveform gives mean arterial pressure (MAP) which indicates perfusion pressure in the vital organs of the body better than SBP [21]. The waveform also can identify the problems that lead to hypertension [22]. For example, the ABP waveform provides information about arterial stiffness which can be optimized for the treatment of high blood pressure [23]. Moreover, the mathematical analysis of this waveform helps to estimate cardiac output [24] and stroke volume [25].

This approach has many advantages.

- Estimating continuous and non-invasive ABP waveform directly from the PPG signal is new and efficient.
- Our proposed method provides SBP, DBP, and MAP values with improved accuracy.
- Our method does not need beat-segmentation of PPG signals
- Non-invasive, continuous, and rapid approach.
- Only PPG signal is required and there is no need to calculate features to estimate BP.
- The proposed U-net model can be trained using a small dataset since it is simple and computationally very efficient.
- The method can be easily applied in a wearable sensor-based device or smartphone.

Our study is organized in the following manner. Section 2 focuses on the relation between the PPG and ABP signal, and our motivation to estimate ABP waveform. Section 3 illustrates the data collection process and the whole method of estimating the ABP waveform using the modified U-net architecture. Section 4 discusses the obtained result and shows a comparison with different methods. Section 5 discusses the limitation of our work and the direction of future research. Finally, Section 6 concludes the paper.

## 2. Motivation

**Figure 1:**
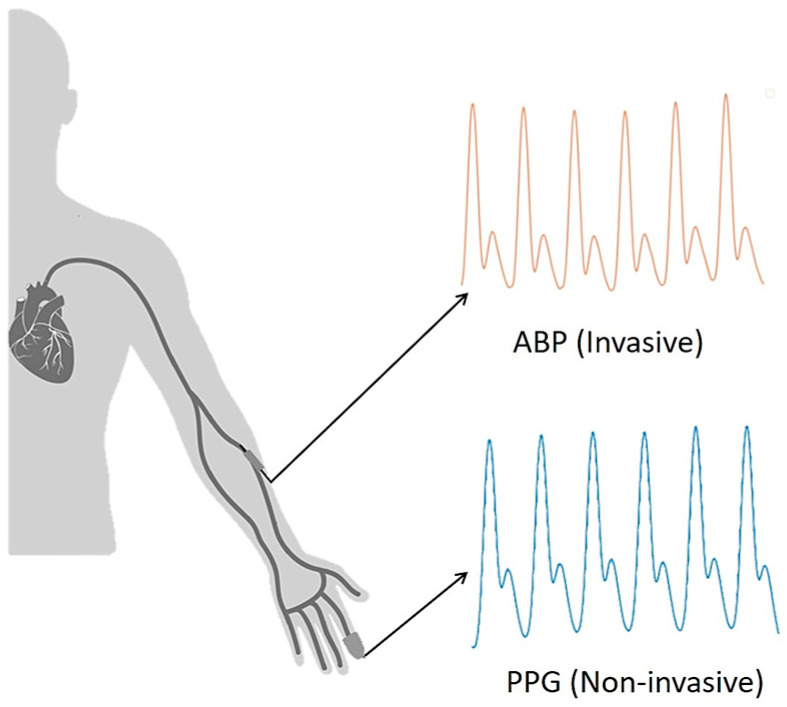
Conventional photoplethysmogram (PPG) and arterial blood pressure (ABP) measurement technique.

The objective of the paper is to estimate the ABP waveform using a PPG signal. Note that PPG can be measured non-invasively using a sensor in the fingertip, while ABP needs to be measured in an invasive way using an arterial catheter. The conventional measurement process of PPG and ABP signal is shown in Figure 1.

When blood flows from the brachial artery to the digital artery, ABP is measured from the brachial artery and PPG from the digital artery [26] as shown in Figure 1. It can be observed that the ABP signal is obtained first and then the PPG signal is measured. So, there exists a path difference between obtained ABP and PPG signals which need to be matched for training our model, and the steps of phase matching will be shown afterward.

The motivation of our work is that a high similarity is observed between the PPG and ABP signals. In Martínez et al. [27], the similarities in time and frequency domains between ABP and PPG signal were analyzed. Pearson’s correlation coefficient between the two signals was higher than 0.9 on average. In Abhay et al. [28], the authors mainly focused on analyzing the feature-based similarity between ABP and PPG signals. The authors considered the features like average slope, peak position, time period, elasticity, and amplitude of the signal. In the research, the peak value of both ABP and PPG signals was observed to occur in a constant time interval. The upstroke time period of both signals was found to have a little difference between 0.02 s to 0.1 s. But the period of heart cycle timing remained the same. Pearson’s correlation coefficient for the elasticity with a peak to peak amplitude of ABP was 0.822. In Tusman et al. [29], 15 cardiac surgery subjects were examined to categorize their BP based on the contour of the PPG signal. The method presented good accuracy 98.4% and 97.8% for detecting episodes of hypotension and hypertension, respectively.

**Figure 2:**
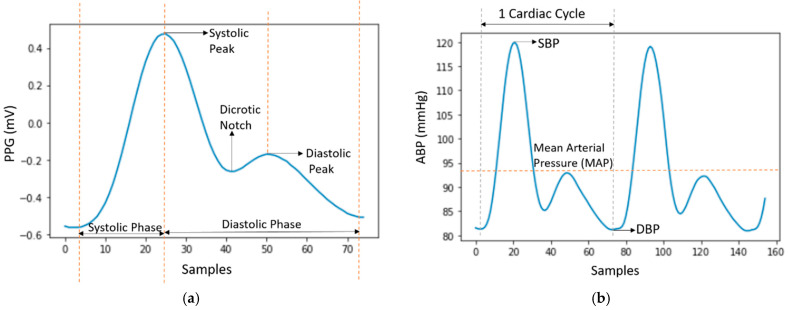
(**a**) Single PPG signal; (**b**) ABP signal with properties.

The properties of these PPG and ABP signals are shown in Figure 2a,b, respectively.

**Figure 3:**
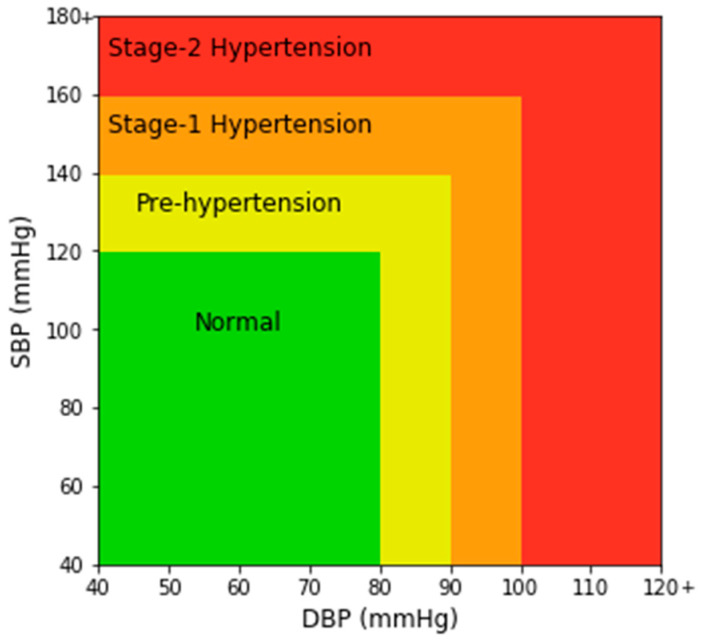
Chart of different blood pressure (BP) ranges.

In Figure 2a, a single PPG signal is shown with a systolic peak, a diastolic peak, a dicrotic notch, and a systolic and diastolic phase. In Figure 2b, the highest peaks denote SBP and the valleys denote DBP. MAP is the average pressure in the arteries during a cardiac cycle. SBP is the maximum pressure within the large arteries when the heart muscle contracts to flow blood through the body. And DBP refers to the lowest pressure within the large arteries during heart muscle relaxation between each beat [30]. In Chobanian et al. [31], four blood pressure ranges of a person are given based on SBP and DBP. The four ranges are normal, pre-hypertension, stage-1 hypertension, and stage-2 hypertension as shown in Figure 3.

## 3. Materials and Methods

**Figure 4:**
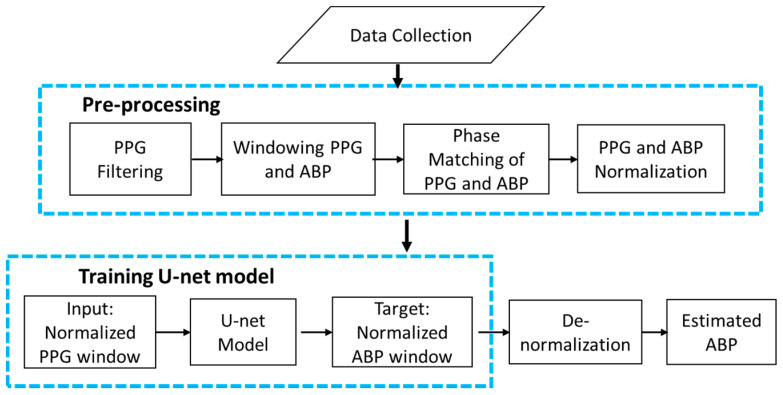
Flow chart of the overall architecture.

The overall architecture of our continuous and non-invasive ABP waveform estimation method is shown using a flow chart in Figure 4. First, the data were collected from databases. Then pre-processing was performed on the obtained data to remove artifacts from the signals. After that, the proposed modified U-net model was trained, validated, and tested with the pre-processed data. Finally, the ABP waveform was obtained from the PPG waveform using the trained U-net model.

### 3.1. Data Collection

The first block of Figure 4 shows the data collection step The invasive ABP and fingertip PPG signals are used for the purpose of the research. The signals are obtained from a combination of two publicly available databases that contain data related to health. One is the MIMIC (Multi-parameter Intelligent Monitoring in Intensive Care) database [32,33] and another is MIMIC-III (Medical Information Mart for Intensive Care III) waveform database [34,35,36]. MIMIC is a publicly available database that contains a myriad of recordings of 121 intensive care unit (ICU) patients. Each recording typically contains between 24 and 48 h of continuous data recorded from patient monitors in the medical, surgical, and cardiac intensive care units of Boston’s Beth Israel Hospital. Another one is the MIMIC-III waveform database, which contains 67,830 records of approximately thirty thousand ICU patients. The databases contain simultaneous recordings of many biological signals, including ABP and fingertip PPG signals with a 125 Hz sampling rate. In this study, we used recordings of 45 subjects of the MIMIC database and 55 subjects of the MIMIC-III waveform database containing both PPG and ABP signals. Ultimately, 100 subjects’ recordings were collected for the experiment, each with complete ABP and PPG signals.

### 3.2. Pre-Processing of Data

The second block of Figure 4 demonstrates the data pre-processing step. Data pre-processing was the most important and time-consuming part of this study. First, PPG data were filtered using the Equiripple FIR filter with cutoff frequencies of 0.5 Hz to 8 Hz [37]. Values below 0.5 Hz were identified as baseline wandering, while values above 8 Hz were considered high-frequency noise.

**Figure 5:**
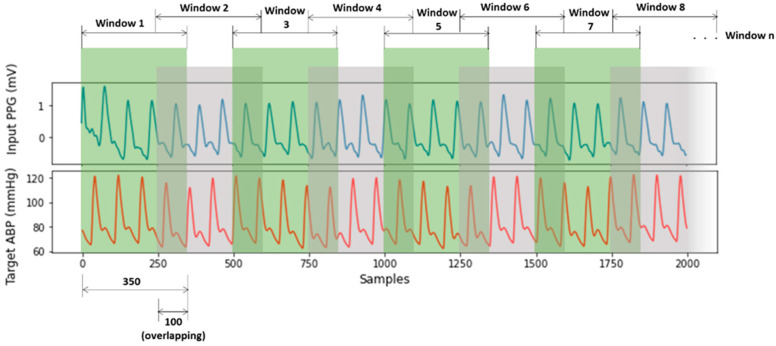
Windowing PPG and ABP signals.

After PPG signal filtering, the PPG and ABP signals were divided into windows of 350 samples sequentially with overlapping of 100 samples in Figure 5. The window size and overlap value were selected empirically, and overlapping was performed to avoid missing information at the boundary of the windows. Windows containing artifacts, that are inaccurate for measurement, were identified and removed using a machine learning model for signal artifact detection [37].

**Figure 6:**
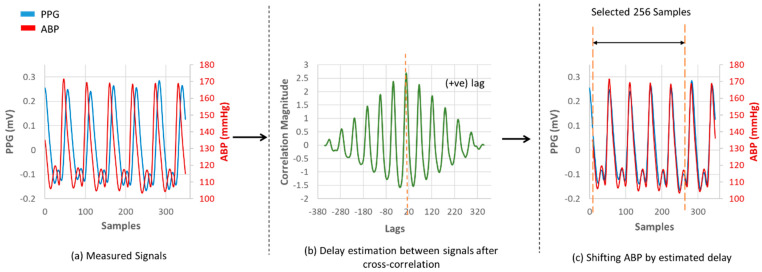
Phase matching between PPG and ABP signals. (**a**) Measured Signals; (**b**) Delay estimation between signals after cross-correlation; (**c**) Shifting ABP by estimated delay.

After that, the ABP and PPG signals were phase-matched according to their phase difference. There occurs a phase difference in PPG and ABP signals due to a path distance as illustrated in Figure 1. This phase difference is considered as a lag in signal analysis. The databases contain signals measured with different devices. So, to match the phase difference between the two signal windows, cross-correlation was performed. The location of the maximum value of the cross-correlation indicates a time lag. Keeping the PPG window fixed, the ABP window was shifted by the estimated time lag. The phase-matching process is shown in Figure 6. As the proposed U-net architecture’s input size is 256 samples, windows having less than 256 samples were not considered for our experiment, and windows having more than 256 samples were trimmed. The duration of each 256 sample window is 2.048 s.

**Figure 7:**
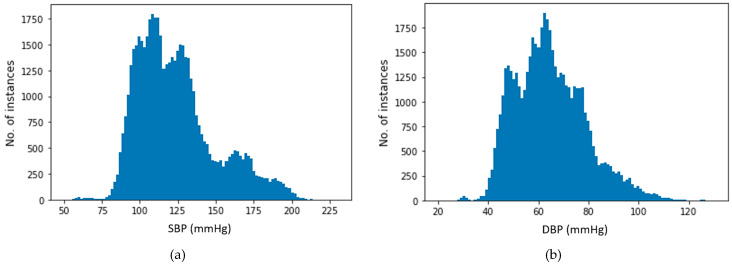
Distribution of (**a**) systolic BP (SBP) and (**b**) diastolic BP (DBP).

The preprocessed dataset was distributed widely from lowest to highest SBP and DBP values. Figure 7 illustrates the distribution of SBP and DBP values used for target values in our estimation process. The plots indicate that all SBP and DBP values for all of the four BP ranges are present in our dataset. After final preprocessing, approximately 195 h of data of 100 subjects were achieved. The final dataset contains on average 3.4 h of data for each subject.

Finally, the obtained data were normalized using Equation (1)

(1) $$x_{normalized\left(i\right)}=\frac{\left(x_{i}-x_{min}\right)}{x_{max}-x_{min}},$$

where *x_i_* refers to *i*-th signal window, and $x_{max}$ and $x_{min}$ are the maximum and minimum values of all the windowed signals, respectively. The normalized PPG and ABP signal windows were used input and target data for the proposed U-net model.

### 3.3. Deep Learning Architecture

**Figure 8:**
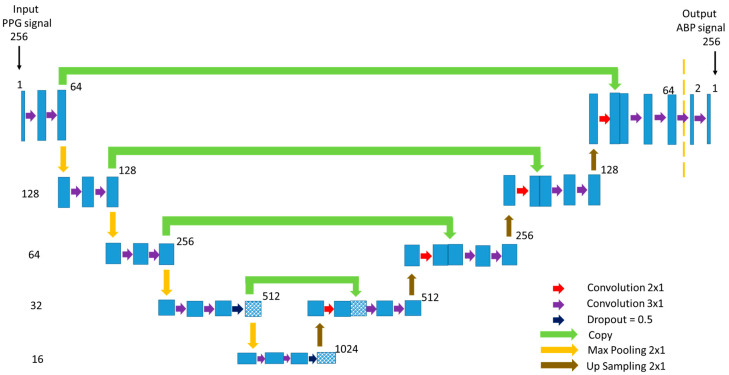
The architecture of the proposed U-net deep learning model.

Our proposed modified U-net model that predicts the non-invasive ABP signal using the PPG signal as input is shown in Figure 8. U-net uses a fully connected neural network model for semantic segmentation [38]. Inspired by this popular U-net architecture, we proposed a modified U-net model. The network architecture is shaped just like a ‘U’ which justifies the name. This architecture consists of three paths: contracting, bottleneck, and expansive path. The contracting path on the left side is made of several contraction blocks and the expansive path is made of several expansion blocks. The bottleneck path intervenes between the contracting path and the expansion path.

In Figure 8, the number on the left side of each block denotes the input vector size of that block. As our goal is to predict a properly shaped ABP signal, the input of the network is a PPG signal with a length of 256 samples of 1 dimensional (1D) convolution layer despite being 2 dimensional (2D) as the actual U-net architecture. In our modified network, each block takes an input that applies two 3 × 1 convolution layers followed by a Leaky Rectified Linear Unit (ReLU) activation function after each convolution layer and 2 × 1 max-pooling layer. The Leaky ReLU activation function was used to avoid the dying ReLU problem. The number of feature channels after each block doubles in the contracting path so that the architecture can learn the complicated structures effectively. The number on the upper corner side of a block denotes the number of feature vectors or channels. The bottleneck path uses two 3 × 1 convolution layers like before followed by a 2 × 1 up-sampling layer. The bottleneck path creates a feature space that automatically generates relevant features to give a proper output signal. Dropout of 50% was applied at the last layers of the contracting path and bottleneck path.

The expansion path is symmetric to the contracting path. Each expansion block passes the input to one 2 × 1 and two 3 × 1 convolution layers followed by a Leaky ReLU activation function after each convolution layer and a 2 × 1 up-sampling layer. After each expansion block, the number of feature channels used by the convolutional layer is halved to maintain symmetry. Moreover, the output of the 2 × 1 convolution layer is concatenated with the feature channels from the corresponding contracting path. This action ensures that the features that are learned while contracting the signal will be used to reconstruct it. Finally, at the last expansion path, two extra 3 × 1 convolution layers are used to map each 64 feature vector equal to the input dimension. Using this network, the PPG signal window of 256 samples is mapped into the ABP signal window of 256 samples. 

### 3.4. ABP Waveform Estimation Process

Using the trained U-net model and a PPG signal as an input to the model, we can estimate an ABP waveform. However, note that we trained the model with normalized PPG signals as input and normalized ABP signals as a target. During the ABP waveform estimation process with the test dataset, we need to use a normalized PPG signal as an input and the output data should be de-normalized. While pre-processing the data, the maximum and minimum values of ABP were saved. They were used as de-normalization factors for the estimated ABP waveform.

SBP, DBP, and MAP were also obtained from the ABP waveform. A standard peak and valley detection algorithm [19] was used to detect the SBP and DBP values of estimated ABP signal windows. For each window, the average of SBP and DBP was taken and considered as the SBP and DBP for that individual window. Similarly, the same algorithm was used to calculate the SBP and DBP values from the ABP windows of the reference test dataset. Generally, in invasive blood pressure measurement, SBP and DBP values are calculated from ABP signal. We utilize the same process to estimate SBP and DBP values from reference and predicted ABP signals to see how much these two values correlate with each other. Additionally, MAP values were obtained by calculating the arithmetic mean of every reference and predicted ABP window [39]. Finally, the obtained values from our model were compared with the reference values for result calculation.

## 4. Performance Evaluation

### 4.1. Hyper-Parameter and Experimental Setup

We used 70% of the total data for training our model, 15% for validation, and the remaining 15% for testing. The training, validation, and test datasets were completely separated from each other. The training dataset is used for network training and depending on the error, the network is tuned. The validation is performed with the validation dataset for generalizing the network and to stop training when generalization does not improve anymore. The testing provides an independent measurement of the network performance. The model was trained using Adam optimizer and the mean squared error loss function was chosen. The learning rate and the batch size were set to 10^−4^ and 4, respectively. The network parameters including the learning rate and batch size were all determined experimentally. Early stopping was used when no improvement was seen in five consecutive epochs and as a result, the training was stopped in 51 epochs. For each epoch in this training process, the epoch performed in lower validation loss was automatically saved. After the model training procedure was finished, the previously auto-saved model was selected as the final trained model. In our experiment, a GPU server containing NVIDIA GTX 1080 Ti 10 GB graphics card and 257 GB system memory was used. All the codes were written in Python.

### 4.2. Predicted Continuous and Non-Invasive ABP Waveform Analysis Results

**Figure 9:**
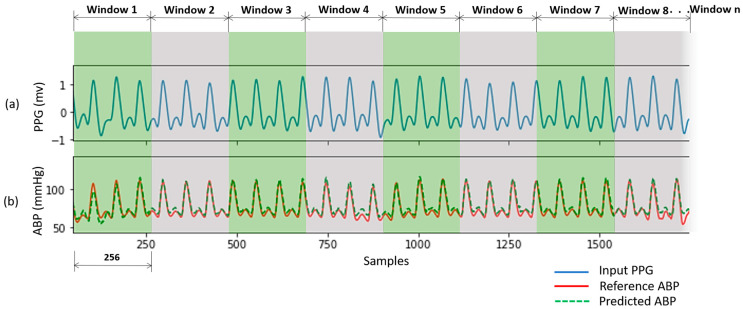
Predicted ABP waveform from PPG of a subject. (**a**) Input PPG signal; (**b**) Reference ABP signal and Predicted ABP signal.

The predicted ABP waveforms using our proposed U-net model correlated highly with the reference waveforms. An example of a subject is shown in Figure 9. The reference ABP signal is measured invasive data but phase matched with the input PPG signal. The predicted ABP waveform is almost accurate as the reference ABP signal. 

**Figure 10:**
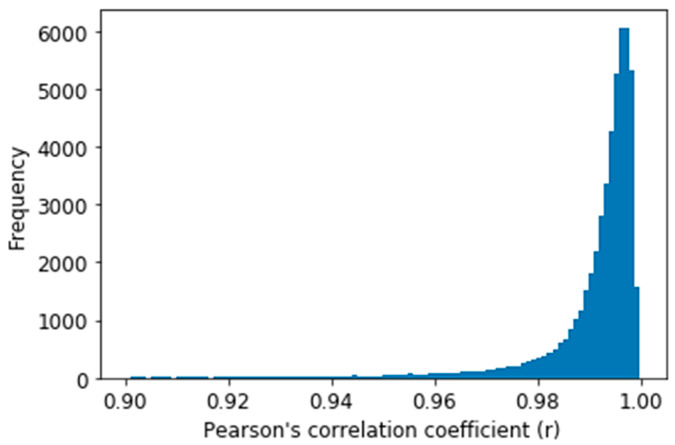
Distribution of Pearson’s correlation coefficient on the test dataset.

To evaluate our obtained ABP waveform result, Pearson’s correlation coefficient (r) was estimated between the predicted and reference ABP signals as this is used to measure the similarity between two-time series data [40]. The distribution of r for all the predicted signal windows in the test dataset is shown in Figure 10. Figure 10 shows that most of the values (X-axis) are between 0.9 to 1.0, which depicts high correlation of the predicted ABP signal with the reference signal windows. And from Table 1, the average Person’s correlation coefficient (r) value can be seen as 0.993. Before calculating the average, we performed Fisher-Z transformation and again performed retransformation after calculating the average to calculate the average r [41]. Table 1 also gives the value of the maximum and the minimum value of r for all the windows. The value of the 25th and 75th percentile of r says that our proposed U-net model predicted most of the waveform accurately. 

### 4.3. SBP and DBP Estimation Results

To evaluate our obtained SBP, DBP, and MAP result, mean absolute error (MAE), standard deviation (STD), root mean square error (RMSE), and Pearson’s correlation coefficient (r) performance metrics were calculated [42]. 

The performance metrics of our proposed model to estimate SBP, DBP, and MAP values are listed in Table 2. The model predicted DBP values comparatively better than SBP values. However, in both cases, the values of measurement factors are quite well which denotes that the proposed model can be used to measure SBP, DBP, and MAP easily and accurately.

**Figure 11:**
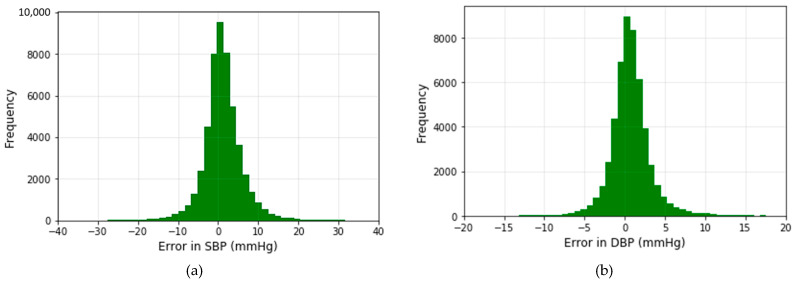
Error histograms of predicted (**a**) and (**b**) DBP values.

The histograms of the prediction error using the modified U-net model for both SBP and DBP values are displayed in Figure 11.

The error is distributed around zero. According to the histogram, the deviation of the predicted value of SBP is almost twice the DBP value. The result recorded in Table 2 proves the correctness of our observation.

**Figure 12:**
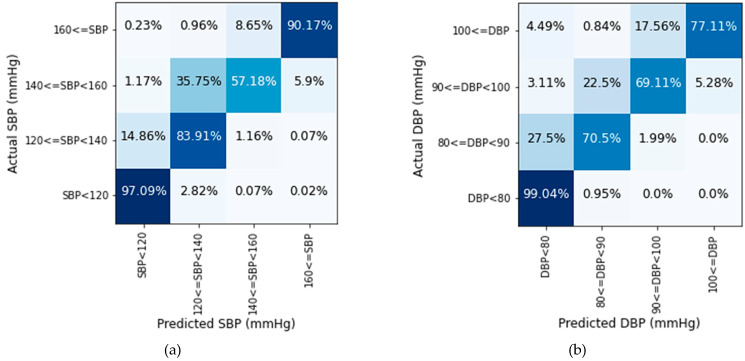
Prediction accuracy of (**a**) SBP and (**b**) DBP values in four BP ranges.

Figure 12 illustrates that the proposed model performs best in the normal BP range. In the normal range, the SBP prediction rate is 97.09% and DBP is 99.04% accurate. In the pre-hypertension range, the prediction of SBP values is better than DBP values for the test dataset. For stage-1 hypertension, the accuracy rate is comparatively low in both cases. In this range, more than 35% of SBP and 22% DBP values deviate more than 10 mmHg. Many values in the range of stage-1 hypertension are identified in the range of pre-hypertension. Though stage-1 hypertension had some deviations in prediction, the prediction rate is quite well for the stage-2 hypertension range. According to Figure 12, the majority of the values are classified properly using the proposed model. The deviation largely remains within 10 mmHg. For a large quantity of data, very few values are found in more than 20 mmHg deviation area which is negligible.

**Figure 13:**
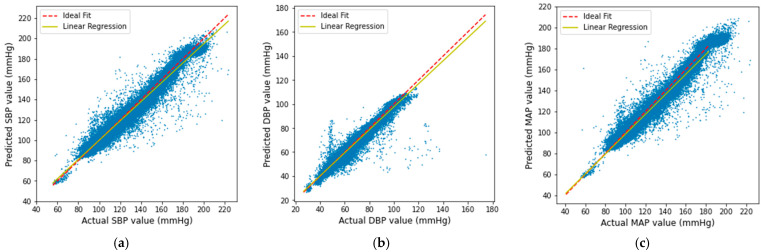
Linear regression plot of the (**a**) SBP, (**b**) DBP, and (**c**) MAP result.

The scatter plots of our predicted result with respect to the actual SBP, DBP, and MAP are shown in Figure 13. It is shown that the obtained result gives a linear correlation, which proves that the predicted result is mostly accurate except in very few cases.

**Figure 14:**
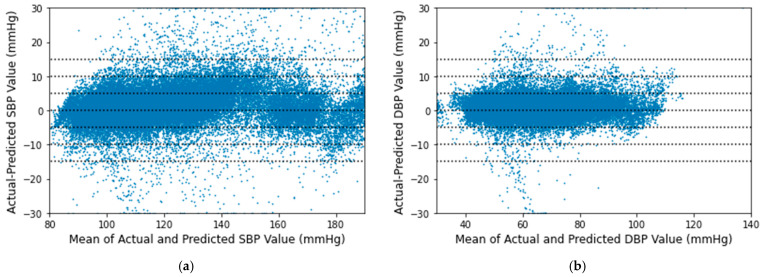
Bland–Altman scatterplot for (**a**) SBP and (**b**) DBP values.

The Bland-Altman plots for the proposed algorithm are displayed in Figure 14 maintaining the AAMI criteria [43]. The x-axes represent pressure from 80 to 190 mmHg for SBP and 30 to 140 mmHg for DBP. The y-axes denote errors in the range of −30 to +30 mmHg. Reference horizontal dotted lines are shown at the interval of 5 mmHg from −15 to +15 mmHg. The mean of each actual BP and its relative predicted BP is plotted across their difference with a point. Greater than 30 mmHg differences are plotted at 30 mmHg and less than −30 mmHg are plotted at −30 mmHg. It is observed that most of the SBP and DBP errors lie between −5 to 5 mmHg in both cases.

### 4.4. Compliance with Standards

The obtained result was compared with the AAMI error range. According to AAMI, the mean difference and standard deviation must be less than or equal to 5 ± 8 mmHg [44]. The predictions of our modified U-net network are fully acceptable for SBP and DBP values. The comparison with the AAMI standard is shown in Table 3.

The accuracy of the model is also checked from the point of view of the British Hypertension Society (BHS) grading standard [45]. The BHS grading standard and the cumulative error percentage of our data are shown in Table 4. According to the result, it can be said that the estimation of SBP and DBP using our model fall in the “Grade A”.

### 4.5. Comparison with Related Works

In general, it is difficult to compare related works due to different evaluation metrics, and the difference in datasets. However, to compare properly, we considered MAE, STD, RMSE, and r as comparison factors. Table 5 summarizes the performance of our proposed method with other related works.

Reviewing other related works, we found Wang et al. [14] used a similar dataset similar to our experiment. They used an artificial neural network (ANN) with their filtered 58,795 single PPG cycles. The filtering process was not clear. They claimed to work with 72 subjects. But in the publicly available MIMIC database among 72 subjects, only 55 subjects have both PPG and ABP signals. In their individual result table, they showed the result of subjects 262, 415, 450 whose either PPG or ABP signal was missing. The signals of subjects 488, 481, 479, 478, and many were absent in the downloaded database, yet they showed their result in the individual table which was confusing. So, these discrepancies undermine the robustness of their conclusions. 

In Xie et al. [15], a very small subset of the Queensland Vital sign dataset was used. The work in Carek et al. [13] and Shimazaki et al. [18] prepared their own dataset which is not publicly available. In Carek et al. [13], two subject-specific parameters were also used to calibrate the data, which will vary for different subjects. However, the studies of Li et al. [12] and Esmaelpoor et al. [47] used the publicly available MIMIC-II database. But Li et al. [12] needed two signals (ECG and PPG) and several features to estimate BP. And on the other hand, our model obtained comparatively better MAE and STD than Esmaelpoor et al. [47]. In Xie et al. [15] and Simjanoska et al. [46], the total amount of data was also very small and the result was also not satisfactory. Moreover, the work of Li et al. [12], Wang et al. [14], Xie et al. [15], Shimazaki et al. [18] and Esmaelpoor et al. [47] needed beat segmentation to detect single PPG cycles.

Another work in Slapničar et al. [16] used waveforms of a huge MIMIC III database. Though they claimed their model could predict regular BP for home use, the result didn’t meet the requirement of the AAMI standard error range.

Compared to the described approaches, our method obtained better accuracy for SBP and DBP values. So our proposed technique can be confidently adopted as the SBP and DBP estimation technique for regularly using devices.

## 5. Discussion

In recent times, a mobile-friendly or wrist watch based reliable BP measurement method is needed to measure BP regularly for a healthy life. So, the proposed model can be used to build a system to measure continuous, non-invasive ABP waveforms and SBP, DBP values easily using smartphones or wearable devices. We focus on estimating the waveform because it is efficient for a 1D-CNN-based model to optimize for a signal target rather than a feature target when the input is a signal. Mainly, the proposed method can be applied for regular use in the current state. It is to be noted that, PPG signals contain different artifacts and the signals are very sensitive to movements, finger pressure, thickness and coloration of the skin, and the nature of the subcutaneous tissue e.g., lots of facts on the fingers. So, this proposed method can be used to monitor the BP of ICU patients if stable PPG signals can be taken using constant pressure on fingertips. However, the problem of artifacts can be overcome. If the detection apparatus is placed at a different location, for example, over the radial and/or ulnar arteries at the wrist, and the patient is instructed to rest and not move the wrist for, say, a period of 30 s each 1 h, then appropriate low noise recordings could be obtained. Moreover, to get accurate measurements for new subjects, a personalized calibration technique can be implemented.

Next, the performance of the deep learning model strongly depends on the quality and amount of data. Our deep learning model used data of ICU patients for training, validation, and testing. The number of stage 2 hypertension subjects were more in the dataset than that of stage 1 hypertension patients. So, our model performed better to predict stage 2 hypertension values than stage 1 hypertension values as shown in Figure 12. Furthermore, the reference ABP waveforms of the databases are measured in the brachial artery. But, our proposed U-net model predicts ABP waveforms from fingertip PPG signal, and this PPG signal is obtained from a digital artery as shown in Figure 1. The predicted ABP waveform is phase-matched with the input fingertip PPG signal as we described before. So, if a person’s ABP waveform is measured from the brachial artery as well as from the digital artery using our system at the same time, then there will be little phase difference between the two signals.

In future studies, there is an opportunity for more research to develop a device that will take stable PPG signals with constant pressure for every critical patient so that it can replace invasive ABP measurement in ICU. Moreover, a real clinical issue is the changes in BP. In the ICU sudden rises or falls in BP are of concern and immediate detection is highly desirable. A prospective study in an ICU can be conducted for checking the sensitivity of changes in BP, particularly wide excursions.

## 6. Conclusions

Continuous, non-invasive, and cuff-less comfortable BP measurement technique is a highly researched topic of the current time, to fight against hypertension. Hypertension is the main cause of several heart-related issues. The development of methods to estimate ABP waveform along with SBP, DBP, and MAP values is a promising yet challenging field. The increasing demand for PPG-based wearable devices can provide an interesting direction in this field as PPG-based technology offers both non-invasive and continuous measurement. Keeping that in mind, our research proposed a U-net deep neural network-based continuous and non-invasive ABP waveform estimation method to detect possible hypertension-based bodily issues at early stages to consider necessary diagnosis steps. Our method gives continuous and non-invasive ABP waveforms that are highly correlated with the reference invasive ABP signals. Moreover, the obtained SBP and DBP values from the predicted ABP waveforms satisfy the AAMI and BHS standard. A complete ABP waveform along with SBP, DBP, and MAP values obtained with our method can help to obtain additional physiological information of a patient and can monitor the BP changes in a better way. We believe, our work can provide a possible solution in the non-invasive, cuffless, continuous measurement methods of BP estimation and assist in keeping BP under control.

## Figures and Tables

**Table 1:** Performance summary of U-net model on test dataset for ABP waveform prediction.

Evaluation Factor	Value
Average r	0.993
Minimum r	0.262
Maximum r	0.999
25th percentile of r	0.989
75th percentile of r	0.996

**Table 2:** Performance summary of proposed U-net Model on test dataset for SBP, DBP, and mean arterial pressure (MAP) values prediction.

Measurement	MAE (mmHg)	STD (mmHg)	RMSE (mmHg)	r
SBP	3.68	4.42	5.75	0.976
DBP	1.97	2.92	3.52	0.970
MAP	2.17	3.06	3.75	0.976

**Table 3:** Comparison of our result with the Association for the Advancement of Medical Instrumentation (AAMI) standard.

		No. of Subjects	MAE (mmHg)	STD (mmHg)
AAMI [44]	BP	>85	≤5	≤8
Our work	SBP	100	3.68	4.42
	DBP		1.97	2.92

**Table 4:** Comparison of the result with British Hypertension Society (BHS) grading standard.

		Cumulative Error (%)		
BHS grading standard [45]		≤5mmH	≤10mmH	≤15mmH
	Grade A	60%	85%	95%
	Grade B	50%	75%	90%
	Grade C	40%	65%	85%
Our work	SBP	76.21%	93.66%	97.71%
	DBP	93.51%	98.70%	99.46%

**Table 5:** Result comparison with related works.

Method	Dataset	Source	Data Used for Testing	SBP|DBP			
				MAE (mmHg)	STD (mmHg)	RMSE (mmHg)	r
Modified U-net (Our work)	MIMIC I, MIMIC III waveform	PPG (raw)	29.3 h	3.68|1.97	4.42|2.92	5.75|3.52	0.976|0.970
ANN [14]	MIMIC I	PPG (feature)	8819 single PPG	4.02|2.27	2.79|1.82	--	--
Random Forest (RF) [15]	Queensland Vital Signs	PPG (feature)	2298 single PPG	4.21|3.24	7.59|5.39	7.57|5.40	0.938|0.942
CNN [18]	Self-made	PPG (raw)	50,000 single PPG	--	11.4	--	0.71
PTT [13]	Self-made	SCG, PPG	--	--	--	4.8|2.9	--
Long short-term memory (LSTM) [12]	MIMIC II	PPG, ECG (feature)	135,641 PPG and ECG cycles	4.63|3.15	14.50|6.44	--	--
Machine Learning [46]	Self-made, Physionet	ECG (feature)	7.8 h	7.72|9.45	--	--	--
ResNet-GRU [16]	MIMIC III Waveform	PPG (raw)	140 h	9.43|6.88	--	--	--
CNN and LSTM [47]	MIMIC II	PPG (raw)	103,760 single PPG	3.97|2.10	5.55|2.84	--	0.95|0.95

## Data Availability

Publicly available datasets were analyzed in this study. This data can be found here: https://physionet.org/content/mimicdb/1.0.0/ (accessed on 7 March 2021) and https://archive.physionet.org/cgi-bin/atm/ATM?database=mimic2wdb&tool=plot_waveforms (accessed on 7 March 2021).
